# A proprietary polyphenolic blend offers improved strength recovery and reduced delayed onset muscle soreness post-exercise

**DOI:** 10.1186/1550-2783-11-S1-P10

**Published:** 2014-12-01

**Authors:** Tatania K Emmick, Kelli A Herrlinger

**Affiliations:** 1 Kemin Foods, L.C., Des Moines, Iowa, USA

## Background

The sports nutrition industry is seeing growth in consumers who are embarking on high intensity daily workout routines to regain and maintain physical fitness. This type of demanding training results in exercise-induced muscle damage setting off a cascade of increased oxidative stress and inflammation which in turn leads to reduced strength and increased delayed onset muscle soreness (DOMS) for days post-training. Consumption of antioxidants and anti-inflammatory molecules is crucial to counteract these negative side effects. A proprietary polyphenolic blend of catechins and theaflavins (PPCT) was formulated and evaluated in a randomized, double-blind, placebo controlled human trial to determine the effect on exercise performance recovery following eccentric exercise.

## Methods

Male participants (age 18-35 years) volunteered and were randomized to receive either a placebo or PPCT (2,000 mg) daily for 13 weeks. During the 13^th^ week of supplementation, subjects performed a 40 minute downhill treadmill run (65% of VO_2max_) with 3 sets of isokinetic leg extension measurements taken at baseline (pre-exercise), 24, 48 and 96 hours post-exercise. Delayed onset muscle soreness (DOMS), muscle damage via creatine kinase (CK), oxidative stress via ferric reducing antioxidant power (FRAP), and a stress hormone (cortisol) were also examined at these timepoints. Consent to publish the results was obtained from all participants.

## Results

The treatment group regained strength as measured by peak torque to 98% and 101% of pre-exercise levels at 48 and 96 hours post-exercise, respectively. In comparison the placebo group’s peak torque levels remained at 92 % and 93% of pre-exercise levels at the same time points post-exercise. These improvements were significant compared to placebo at both time points (p < 0.05). Additionally, participants in the PPCT group reported decreased whole body and hamstring DOMS compared to placebo at 48 hours (p = 0.029 for both). These enhancements in strength and DOMS were also supported by improvements in serum markers of oxidative stress, muscle damage and inflammation. Chronic consumption of PPCT improved serum antioxidant status (p=0.039) as measured by FRAP. As expected, serum cortisol increased in all groups compared to pre-exercise levels; however by 96 hours, serum cortisol levels had returned to pre-exercise levels in the PPCT group while the placebo remained 20% above pre-exercise levels (p < 0.05). Creatine kinase (CK) increased in both groups peaking at 24 hours post-exercise. CK levels returned to pre-exercise values at 96 hours in the PPCT groups while levels in the placebo group remained significantly elevated 50% over pre-exercise levels (p < 0.05) at the same time point. These reductions in cortisol and CPK levels occur simultaneous to the recovery in pre-exercise strength observed at 96 hours.

## Conclusions

Daily supplementation with PPCT was shown to reduce DOMS and promote recovery of muscle strength by reducing the oxidative stress and markers of muscle damage that occurs post-exercise.

